# β-blockades and the risk of atrial fibrillation in patients with cardiovascular diseases

**DOI:** 10.3389/fphar.2024.1418465

**Published:** 2024-06-25

**Authors:** Xun-Hu Gu, Weichao Li, Heng Li, Xun Guo, Jiang He, Yuyan Liu, Jianping Gong, Yizhou Huang, Bin Zhang

**Affiliations:** ^1^ Department of Neurology, The Second Affiliated Hospital of Nanchang University, Nanchang, China; ^2^ Department of Anesthesiology, Affiliated Qingyuan Hospital, Qingyuan People’s Hospital, Guangzhou Medical University, Qingyuan, China

**Keywords:** β-blockers, atrial fibrillation, risk, NHANES (national health and nutrition examination survey), cardiovascular diseases

## Abstract

**Background:**

β-blockers have been widely used in patients with extensive cardiovascular disease (CVD) and have provided benefits. However, they are more likely to cause symptomatic bradycardia, hypotension, or glucose metabolism disorders, which may lead to an increased risk of atrial fibrillation (AF), but evidence is lacking.

**Aims:**

This study was to analyze the association between the use of β-blockers and the risk of developing AF.

**Methods:**

This nationwide, prospective cohort study utilized data from the 2013–2020 National Health and Nutrition Examination Survey (NHANES). The patients were stratified into a β-blocker treatment group (*n* = 2585) and a non-β-blocker treatment group (*n* = 8525). Univariate and multivariate logistic regression analyses were performed to identify the relationship between β-blockades and the risk of AF. Propensity matching analysis was used to balance patient baseline characteristics and to control for confounders.

**Results:**

A total of 11,110 subjects were included in this study (mean [SD] age, 59.89 [15.07] years; 5657 [49.7%] males). A total of 111/2585 subjects developed AF in the β-blocker treatment group, and 75/8525 developed AF in the non-β-blocker treatment group (incidence rate, 4.2% vs. 0.8%). Compared with the non-β-blocker group, the β-blocker group had an increased risk of incident AF (aOR, 2.339; 95% CI, 1.614–3.410). Some sensitivity analyses also revealed consistent findings of increased AF risk associated with β-blocker treatment.

**Conclusion:**

The findings from this study suggest that β-blocker treatment is associated with an increased risk of incident AF and may help physicians select a modest medication for patients while also assessing the risk of AF.

## Graphical Abstract

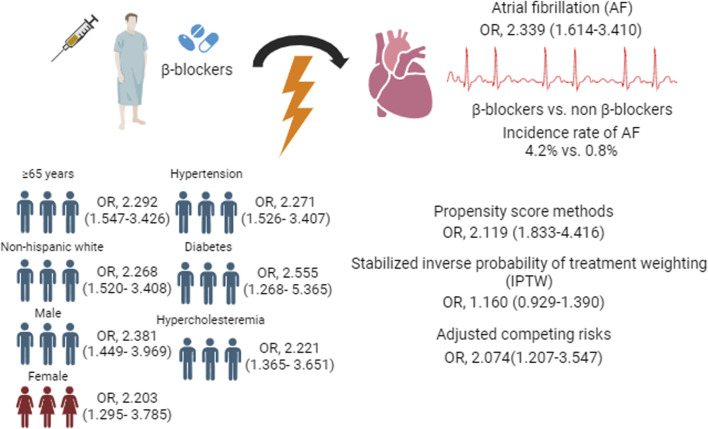

## Introduction

Beta-adrenergic receptor blockers (β-blockers) were first used to treat angina in 1960 and have since been widely used to treat cardiac diseases. Previously, several large, randomized controlled trials showed reductions in mortality and morbidity with β-blocker treatment in subjects with heart failure with reduced ejection fraction (HFrEF) (Cleland et al., 2018). However, based on emerging evidence, the benefit of β-blockers in patients with heart failure (HF) is controversial because β-blocker use in heart failure patients with a modest or preserved ejection fraction (HFmEF or HFpEF) was already associated with a greater risk of HF hospitalization in a large, real-world, propensity score-adjusted cohort (Arnold et al., 2023). In general, atrial fibrillation (AF) is a comorbidity of HF and is associated with increased morbidity and mortality. Swift conduction of AF to the ventricles is a frequent cause of HFpEF. The main atrioventricular node-suppressing drugs used are β-blockers, which are strongly recommended by the guide. However, controversy remains. Two large randomized controlled studies suggested that β-blockers reduced the functional capacity and increased the levels of NT-proBNP compared with digoxin or nondihydropyridine calcium channel blockers in patients with permanent AF (Kotecha et al., 2020; Ulimoen et al., 2014). Some side effects of β-blockers that may predispose patients to AF, such as bradycardia, worsening glycemic control, and new-onset diabetes mellitus, have recently been highlighted (Bangalore et al., 2007; Elliott and Meyer, 2007). In the AFFIRM trial randomized at a 1:1 ratio, bradycardia during sinus rhythm occurred in 17% of the subjects in the nondihydropyridine calcium channel blocker group compared with 32% in the β-blocker group (Koldenhof et al., 2023). Additionally, patients randomized to receive atenolol showed strongly increased central blood pressure, which is a biomarker of AF (Williams et al., 2006). β-blockers affect lipid and glucose metabolism, which may also play a role in increasing the risk of AF. Previous reviews have also suggested that beta-blockers may increase the risk of AF in patients who are in sinus rhythm, but there is a lack of hard evidence (Meyer and Lustgarten, 2023). On the basis of the above data, we hypothesized that β-blockers may increase the risk of AF in patients with sinus rate.

## Methods

### Study population

A sophisticated and intricate methodology, the NHANES database, collected representative US population sample information at 2-year intervals. The intense goal of this database is to analyze and identify individuals’ health and nutritional status in the US. Approval for the NHANES protocols was obtained from the National Center for Health Statistics Institutional Review Board, and each subject provided written informed consent. The NHANES covers several columns of data, including demographic data, daily diet data, medical detection data, laboratory test data, and questionnaire data. We included 4 survey cycles from 2013 to 2020. Patients with cardiovascular diseases, such as hypertension, diabetes mellitus, or hypercholesteremia, were included in this study. After these exclusions, the present study included a total of 11,110 patients aged 18 years or older. The trial was reported in agreement with the Strengthening the Reporting of Observational Studies in Epidemiology (STROBE) reporting guidelines and The Reporting of Studies Conducted Using Observational Routinely-Collected Health Data (RECORD) statement (von Elm et al., 2007; Benchimol et al., 2015).

### Covariate

The NHANES protocols from 2013 to 2020 obtained information on several factors from subjects, including demographic data (including age, sex, BMI, race, smoking habits, alcohol consumption status, insurance status, and employment status), comorbidities (including hypertension, diabetes mellitus, and hypercholesteremia), and therapeutic medication (including β-blockers, CCB, ARB/ACEI, diuretics, insulin, metformin, and statins). The BP of well-trained staff members was measured with a mercury sphygmomanometer following the case, and the staff members rested fully in a seated position for 5 min. A systolic blood pressure (SBP) greater than 140 mmHg and/or diastolic blood pressure (DBP) greater than 90 mmHg were considered to indicate hypertension, and subjects receiving antihypertensive medicine were also considered to have hypertension (Whelton et al., 2017). A fasting blood glucose concentration greater than 7 mmol/L (≥126 mg/dL) and/or a hemoglobin Alc concentration exceeding 6.5% were considered indicative of diabetes (ElSayed et al., 2023). A ratio of total cholesterol to high-density lipoprotein cholesterol greater than 5 was considered to indicate hypercholesterolemia (Expert Panel on Detection et al., 2001). The main outcome of the study was atrial fibrillation (code I48), according to codes from the International Classification of Diseases, 10th Revision. The diagnostic codes used to confirm relative covariates are provided in Supplementary Table S3.

### Statistical analysis

The sample size calculation was based on a prevalence of AF of approximately 1.6% determined by the 2013–2020 CVD cohorts. We expected to enroll approximately 1000 patients with CVD and detect 17 patients with AF. Considering that the C-statistic was set to 0.8 and the number of candidate predictor parameters was set to 24, at least the sample size required to develop a model would need 9480 subjects, assuming an acceptable difference of 0.05 in the apparent adjusted R2 and a margin of error in estimating the intercept of 0.05 (Riley et al., 2020).

The study used the mean (±SD) to express continuous variables, which were compared using a *t*-test or Wilcoxon rank-sum test based on the results of the Kolmogorov‒Smirnov normality test. Categorical variables are expressed as frequencies (percentages) and were compared using the chi-squared test. To determine the association between the use of β-blockers and the risk of AF, both univariable and multivariable-adjusted logistic regressions were used to calculate odds ratios (ORs) with 95% confidence intervals (CIs). Propensity matching analysis was conducted to balance the baseline diversities and to assess underlying confounding variables. A propensity score was primarily computed for each subject to assess the odds of being distributed to the β-blocker group by using multivariable logistic regression models on the basis of all the covariates outlined in Supplementary Table S2. The propensity score was subsequently calculated for the β-blocker and non-β-blocker groups at a 1:2 ratio (Parsons LS Ovation Research Group, 2001).

Some subgroup analyses were conducted, including those classified by older age (<65 and ≥65 years), sex, race, and the presence of hypertension, diabetes, and hypercholesteremia. Diversity sensitivity analyses confirmed the stability of our results. First, the propensity score matching method was used to balance confounding variables in the sensitivity analysis (1:2 ratio). Second, stabilized inverse probability of treatment weighting (IPTW), the second propensity score method, was used to control for confounders (Desai and Franklin, 2019). Third, we performed a sensitivity analysis by adjusting for various combinations of antihypertensive medications in the multivariate logistic regression analysis.

A 2-sided *p*-value less than 0.05 was considered to indicate statistical significance, and all the statistical analyses were performed using SPSS version 25 (IBM) and R 4.2.1, which are based on the R Foundation.

## Results

**FIGURE 1:**
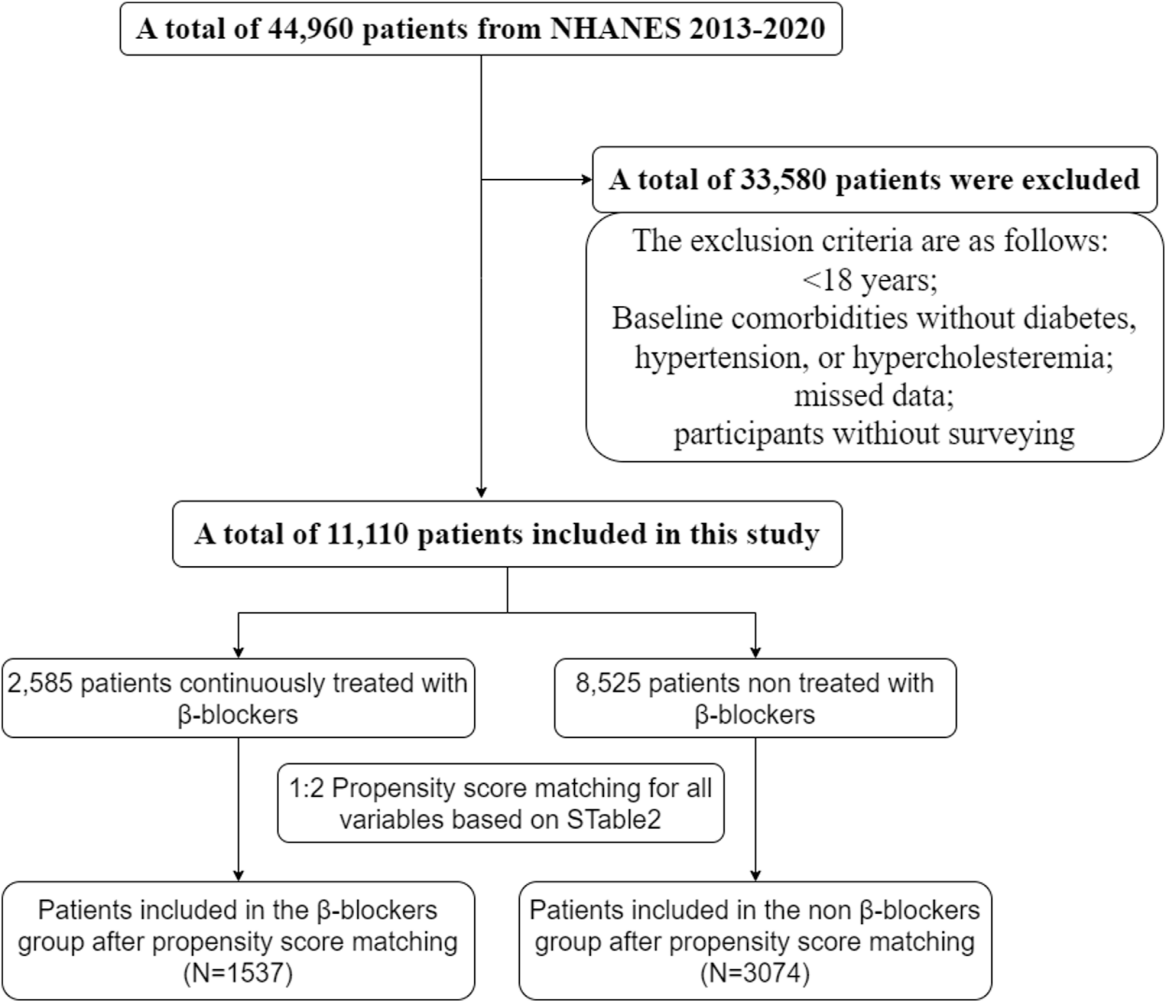
Screening flowchart.

We screened NHANES data from 2013 to 2020, and a total of 11,110 patients were enrolled in the study. The exclusion criteria were as follows: <18 years, no diabetes, hypertension, or hypercholesteremia at baseline, missing data, and participated in the survey (Figure 1).

### Participant characteristics

The features of the study subjects are shown in Table 1. In general, β-blocker populations had greater proportions of risk factors than non-β-blocker populations, which included age, race, BMI, smoking status, drinking status, noninsurance status, unemployment status, comorbidities, and medications. Patients who used β-blockers had a greater incidence of atrial fibrillation than did those who did not use β-blockers. Supplementary Table S1 also outlines the population characteristics of AF patients and non-AF patients. The AF cohort had greater proportions of older, non-Hispanic white person, smoking, drinking, unemployment, cardiovascular disease, and medication use than did the non-AF cohort.

**TABLE 1:** Baseline characteristics of the β-blocker and no β-blocker groups.

	Overall	β-blockers	Non β-blockers	*p*-Value
Covariates	*n* = 11110	*n* = 2585	*n* = 8350	
Age (mean (SD))	59.94 (15.02)	66.33 (11.87)	58.00 (15.33)	<0.001
Male (%)	5503 (49.5)	1266 (49.0)	4237 (49.7)	0.532
Race (%)				
Mexican American	1224 (11.0)	211 (8.2)	1013 (11.9)	<0.001
Other Hispanic	1031 (9.3)	223 (8.6)	808 (9.5)	
Non-Hispanic White people	4270 (38.4)	1198 (46.3)	3072 (36.0)	
Non-Hispanic Black people	3078 (27.7)	686 (26.5)	2392 (28.1)	
Other Race	1507 (13.6)	267 (10.3)	1240 (14.5)	
BMI (mean (SD))	21.67 (5.34)	21.81 (5.50)	21.63 (5.29)	0.129
Obesity (%)	756 (6.8)	194 (7.5)	562 (6.6)	0.117
Smoking (%)				
Never	5610 (50.5)	1298 (50.2)	4312 (50.6)	0.836
Former	3221 (29.0)	746 (28.9)	2475 (29.0)	
Now	2279 (20.5)	541 (20.9)	1738 (20.4)	
Drinking (%)	7372 (66.4)	1610 (62.3)	5762 (67.6)	<0.001
Insurance (%)				
No	426 (3.8)	58 (2.2)	368 (4.3)	<0.001
Yes	9884 (89.0)	2443 (94.5)	7441 (87.3)	
Other	800 (7.2)	84 (3.2)	716 (8.4)	
Employment				
Working at a job	4386 (39.5)	622 (24.1)	3764 (44.2)	<0.001
With business but not at work	193 (1.7)	27 (1.0)	166 (1.9)	
Looking for work	241 (2.2)	29 (1.1)	212 (2.5)	
Not working	6281 (56.5)	1905 (73.7)	4376 (51.3)	
Other	9 (0.1)	2 (0.1)	7 (0.1)	
Diabetes (%)	3750 (33.8)	969 (37.5)	2781 (32.6)	<0.001
Hypertension (%)	9816 (88.4)	2412 (93.3)	7404 (86.9)	<0.001
Hypercholesteremia (%)	6121 (55.1)	1670 (64.6)	4451 (52.2)	<0.001
Taken medicine in past month (%)				<0.001
Yes	9666 (87.0)	2585 (100.0)	7081 (83.1)	
No	1437 (12.9)	0 (0.0)	1437 (16.9)	
Refused	5 (0.0)	0 (0.0)	5 (0.1)	
Other	2 (0.0)	0 (0.0)	2 (0.0)	
Medicines taken, No. (median [IQR])	4.00 [2.00, 6.00]	6.00 [4.00, 8.00]	3.00 [1.00, 5.00]	<0.001
CCB (%)	3750 (33.8)	969 (37.5)	2781 (32.6)	<0.001
ARB/ACEI inhibitor (%)	2271 (20.4)	660 (25.5)	1611 (18.9)	<0.001
Diuretic (%)	5322 (47.9)	1354 (52.4)	3968 (46.5)	<0.001
Statins (%)	2349 (21.1)	780 (30.2)	1569 (18.4)	<0.001
Insulin (%)	3524 (31.7)	1135 (43.9)	2389 (28.0)	<0.001
Metformin (%)	1432 (12.9)	492 (19.0)	940 (11.0)	<0.001
AF (%)	186 (1.6)	111 (4.2)	75 (0.8)	<0.001

The use of medicine in the past month was defined as the use or use of medication for which a prescription was given in the past 30 days.

Medicines taken were defined as the number of prescription medicines reported.

Abbreviations: AF, atrial fibrillation; CCB, calcium channel blocker; ARB, angiotensin receptor blocker; ACEI, angiotensin converting enzyme inhibitor.

After the propensity score matching process, a total of 4611 patients were included in the analysis. The mean (SD) age of the participants was 63.6 (12.3) years, and there were 2416 (52.4%) women and 2195 (47.6%) men. Among the included patients, 1537 were in the β-blocker group, and 3074 were in the non-β-blocker group. All baseline features were fine matched between the two groups, with standardized differences <0.1 for all baseline covariates (as shown in Supplementary Table S2).

## Risk of developing AF

The multivariable logistic regression model indicated that β-blocker treatment was associated with a greater risk of incident AF (adjusted OR, 2.339; 95% CI, 1.614–3.410) (Table 2). A total of 111 subjects developed AF in the β-blocker group, and 75 subjects developed AF in the non-β-blocker group (incidence rates, 4.2 vs. 0.8).

**TABLE 2:** Risk of AF development in the β-blocker and Non β-blocker groups^a^.

	β-blocker group (*n* = 2585)	Non β-blocker group (*n* = 8525)
AF event, No.	111	75
Incidence rate	4.2%	0.8%
Adjusted OR (95% CI)^b^	2.339 (1.614–3.410)	1 [Reference]

^a^ The β-blocker group included subjects durative to receive β-blocker; subjects in the counter group to little receive the drug.

^b^ Calculated using the multivariable logistics regression model.

Abbreviation: OR, odd ratio.

### Stratified analyses

Table 3 and Supplementary Table S4 show the results of the stratified analyses. Among subjects ≥65 years, the risk of AF was significantly greater in the β-block group than in the no β-block group (adjusted OR, 2.292; 95% CI, 1.547–3.426); however, this difference also appeared robust in subjects <65 years (adjusted OR, 5.782; 95% CI, 2.167–16.540). A greater risk of AF associated with β-block treatment was detected in both the male (adjusted OR, 2.381; 95% CI, 1.449–3.969) and female (adjusted OR, 2.203; 95% CI, 1.295–3.785) cohorts. Similarly, a greater risk of AF was found in non-Hispanic white persons and in other ethnic populations. An increased risk of AF in patients treated with β-blockers was also found regardless of the presence of the following comorbidities: hypercholesteremia (with hypercholesteremia: adjusted OR, 2.221 [95% CI, 1.365–3.651]; without hypercholesteremia: adjusted OR, 3.750 [95% CI, 2.039–7.123]), hypertension (with hypertension: adjusted OR, 2.271 [95% CI, 1.526–3.407]; without hypertension: adjusted OR, 3.750 [95% CI, 2.039–7.123]), and diabetes (with diabetes: adjusted OR, 2.555 [95% CI, 1.268–5.365]; without diabetes: adjusted OR, 2.231 [95% CI, 1.435–3.485]). Supplementary Table S5 show the results of analysis of the relationship between types of β-blocker and AF.

**TABLE 3:** Risk of AF development between β-blockers and Non β-blocker groups in the subgroup.

Population	Adjusted OR (95% CI)	*p*-Value
Overall population	2.339 (1.614–3.410)	<0.001
Stratification by age-y		
≥65	2.292 (1.547–3.426)	<0.001
<65	5.782 (2.167–16.540)	0.001
Stratification by race		
Non-hispanic white people	2.268 (1.520–3.408)	<0.001
Other races	4.171 (1.684–11.326)	0.003
Stratification by sex		
Male	2.381 (1.449–3.969)	0.001
Female	2.203 (1.295–3.785)	0.004
Stratification by hypertension		
Hypertension	2.271 (1.526–3.407)	<0.001
No hypertension	3.750 (2.039–7.123)	<0.001
Stratification by diabetes		
Diabetes	2.555 (1.268–5.365)	0.01
No diabetes	2.231 (1.435–3.485)	<0.001
Stratification by hypercholesteremia		
Hypercholesteremia	2.221 (1.365–3.651)	0.001
No hypercholesteremia	3.750 (2.039–7.123)	<0.001

### Sensitivity analyses

The sensitivity analyses included propensity score matching (1:2 ratio) with logistic regression models (adjusted OR, 2.119; 95% CI, 1.547–3.426) and stabilized IPTW (adjusted OR, 1.160; 95% CI, 0.929–1.390) to adjust for competitive confounding variables, and the results were consistent with our analyses. The sensitivity analysis considering β-blockers plus CCB, β-blockers plus ARB/ACEI, and β-blockers plus diuretics as competing risk factors also showed analogous findings (adjusted OR, 2.074; 95% CI, 1.207–3.547) (Table 4).

**TABLE 4:** Sensitivity analyses to assess risk of af development in the β-blocker and No β-blocker groups.

	Adjusted OR (95% CI)	*p*-Value
Multivariable regression models with propensity score methods^a^		
β-blocker group	2.119 (1.205–3.731)	0.009
No β-blocker group	1 (Reference)	
Stabilized IPTW^b^		
β-blocker group	1.160 (0.929–1.390)	<0.001
No β-blocker group	1 (Reference)	
Adjusted competing risks^c^		
β-blocker group	2.074 (1.207–3.547)	0.008
No β-blocker group	1 (Reference)	

^a^ Calculated using the multivariable logistics regression mode in the propensity score-matched population.

^b^ Using stabilized IPTW, instead of propensity score matching to control for potential confounding effects.

^c^ Adjusted for the use of other dual antihypertensive medications (e.g., β-blocker plus CCB, β-blocker plus ARB/ACEI, and β-blocker plus diuretic).

## Discussion

In this large-scale, prospective, national cohort study, subjects who received sustainable treatment with β-blockers for cardiovascular disease had a significantly greater risk of developing AF than patients who did not receive β-blockers, suggesting a potential association between β-blockers and increased AF risk. Some comorbidity-classification analyses revealed that an increased risk of AF was also associated with β-blockers. These findings were consistent with some sensitivity analyses, demonstrating the steadiness of the results.

To date, clinical evidence about the effect of β-blockers on AF risk is very limited. Two large, randomized antihypertensive trials suggested that β-blockers may increase the risk of AF. In the LIFE blood pressure lowering trial, the use of a beta-blocker increased the risk of AF >30%, with the primary outcome of a comparison of losartan with atenolol. In the ASCOT antihypertensive trial, beta-blockers (atenolol) still play a side role in increasing the risk of malignant arrhythmia compared with amlodipine. The combination of antihypertensive agents may confound the primary outcome in the ASCOT trial, and as a secondary outcome, there may not be a significant difference between the two arms for life-threatening arrhythmias. The LIFE trial, which lacked controls or other antihypertensive drugs, did not suggested that atenolol is an independent risk factor for atrial fibrillation. Thus, there is no direct evidence to support an association between β-blockers and AF. Therefore, we conducted a nationwide, prospective cohort study to assess whether the continuation of β-blocker use increased the risk of AF.

According to our comorbidity analysis (Table 2), β-blocker use in patients with diabetes, hypertension, or hypercholesteremia was associated with an increased risk for AF. Tetsuro et al. suggested that in the multicountry and multicenter ACCORD trial with propensity score matching, the use of β-blockers increased the risk of cardiovascular events, including nonfatal myocardial infarction, unstable angina, nonfatal stroke, and cardiovascular death, in subjects with diabetes mellitus (Tsujimoto et al., 2017). Jay S et al., for a randomized trial in diabetic patients, also suggested that baseline β-blocker use increased the risk of primary cardiovascular outcomes compared with no β-blocker use (Shavadia et al., 2019). Additionally, β-blocker use in the general population is associated with an increased risk of insulin resistance, worsening glycemic control, and new-onset diabetes (Elliott and Meyer, 2007; Stump et al., 2006), and these factors may also induce atrial fibrillation. Our findings are also consistent with the above findings; similarly, a greater proportion of patients in the β-blocker treatment group were treated with antihyperglycemic agents and had diabetes mellitus. β-blockers may increase the risk of AF in the hypertensive population. β-blockers cannot benefit patients from hypertension-related cardiovascular complications and may even increase the risk of stroke or death (Dahlöf et al., 2002). Some antihypertensive drugs are not recommended as first-line antihypertensive drugs for the US population. Based on the close interaction between AF and stroke or other adverse cardiovascular events, it may also be plausible that beta-blockers increase the risk of AF in hypertensive patients. β-blockers affect lipid metabolism. George L et al., for a large sample randomized controlled trial, suggested that metoprolol increased triglycerides, whereas carvedilol had no effect (Bakris et al., 2004). Rhonda M et al. also obtained similar findings in randomized hypertensive patients with and without abdominal obesity (Cooper-DeHoff et al., 2010). According to our findings, a greater proportion of patients in the β-blocker treatment group were taking lipid-lowering drugs and had hypercholesteremia. Dysregulated lipid metabolism is associated with an increased risk of atrial fibrillation (Pellegrini et al., 2023).

Heart failure (HF) with reduced ejection fraction (LVEF below 40%) is the only indication where selective β-blockers have a marked benefit, improving ejection fraction and prolonging life. The moderate advantages of β-blockers after myocardial infarction (MI) were only evident in the time before revascularisation when patients had bigger MIs and decreased ejection fractions. An initial indication that the benefits of β-blockers were ejection fraction dependent emerged from randomized trials of β-blockers in the rapid reperfusion setting, which showed an unexpected increase in heart failure hospitalizations with β-blockers. Several articles have been published that also confirmed that in patients with an EF of 50% or more, β-blockers use was associated with an increased risk of HF hospitalization, but not CVD mortality. There was no such association in patients with an EF between 45% and 49% (Silverman et al., 2019; Arnold et al., 2023). β-blockers may not benefit patients with heart failure combined with AF. In a meta-analysis of 4 trials involving 8,680 patients with HF, Michiel et al. found that the effect of beta-blockers on outcome was less in HF patients with reduced systolic LVEF who had AF than in those with sinus rhythm (Rienstra et al., 2013). Current guidelines for the treatment of AF also recommend that rhythm-controlling drugs should be used first, rather than rate-controlling drugs, because patients who use rhythm-controlling drugs first have a better prognosis (Camm et al., 2022; Parkash et al., 2022).

The female hormones may also influence beta-blocker-caused AF. Despite the differences in risk, etiology, and prognosis of HF between men and women, current guidelines do not differentiate between the use of beta-blockers in men and women (Pina, 2003). The Framingham study showed that hypertension has the greatest impact on the risk of HF, accounting for 39% of HF in men and 59% in women (Yusuf et al., 2004). In addition, hypertension is more strongly associated with coronary heart disease in women than in men, and women have a higher risk of HF after myocardial infarction. Clinical outcomes do not improve to the same extent in women with HF as in men (Cenko et al., 2019). The potentially harmful effect of β-blockers was magnified by some clinical presentations. Women with ST-elevation MI had a 6.1% absolute higher rate of HF compared with men, while the possible adverse impact of previous β-blocker use in women was not observed in non-ST-elevation acute coronary syndrome (ACS). HF was also associated with an increased risk of AF in both women and men. A potential mechanism for the greater risk of HF in women may be an interplay between hormone replacement therapy and β-blockers. Progestins may block the cardiac presentation of beta-1-adrenergic receptors and decrease beta-adrenergic-mediated excitation, which may reduce cardiac output and increase susceptibility to HF. Given the close association between HF and AF, gender differences in the triggering of AF by beta-blockers are well documented.

This study could not identify the latent biological mechanisms underlying the association between β-blockers and an increased risk of AF; however, several previous reports may help analyze these results. β-blockers extend the diastolic filling interval by reducing the heart rate. As described in previous studies, prolonged diastolic filling resulting from BB induces greater filling pressure because the extra blood volume is necessary to overcome the rising resistance of the dilating ventricle (Coltart et al., 1975). Accordingly, higher filling pressures stretch left atrial and ventricular wall tension. Increased chronic atrial afterload is detrimental to atrial function and sparks atrial remodeling and hypertrophy. In addition, beta-blockers reverse intraheart hyperemia in response to concomitant increases in brain natriuretic peptide levels, a biomarker of atrial and ventricular wall stretching that predicts AF (Hamatani et al., 2021). β-Blocker use may induce abnormalities in glucose metabolism (Lithell, 1991). Clinical studies have shown that abnormal glucose metabolism can lead to proarrhythmic electrophysiologic changes. Patients with abnormal glucose metabolism were shown to have prolonged atrial activation times and lower bipolar voltages during catheter ablation (Chao et al., 2010), indicating the occurrence of proarrhythmic electrical remodeling. Additionally, abnormal glucose metabolism may also affect atrial excitation-contraction coupling, leading to increased atrial fibrosis, interatrial conduction disorders, and a decreased threshold for AF (Fu et al., 2013). Furthermore, subjects with impaired fasting glycemia were observed to have prolonged conduction times, along with reductions in left atrial voiding volume and evacuation fraction (Ayhan et al., 2012). Similarly, the incidence of interatrial and intra-atrial electromechanical disorders was significantly greater in patients with diabetes than in healthy control patients (Demir et al., 2016). Furthermore, proarrhythmic electrical remodeling, prolonged conduction times, and atrial excitation-contraction uncoupling occurred on the basis of disturbances in cardiac ion channels including inward rectifier K^+^-current upregulated, Ca^2+^-handling dysregulation, and increased sarcoplasmic-reticulum (SR) Ca^2+^-content and cardiac ryanodine-receptor channel type-2 (RyR2) expression (Nattel et al., 2020). The imbalance in sympathetic and parasympathetic activity caused by abnormal glucose metabolism may also contribute to the development of AF (Kuehl and Stevens, 2012).

### Strengths and limitations

The strengths of the NHANES include the use of a nationally representative sample of multiethnic populations of various age groups; the systematic collection of demographic, comorbidity, occupation, income, and prescription medication variables with standard methods and stringent quality control; and the use of primary outcome codes from the International Classification of Diseases, 10th Revision. This study also has several limitations. First, due to the nonrandomized nature of the subject population, there is certainly some bias in the study. Randomized controlled trials are urgently needed in the future. Second, we do not know the type of AF, such as paroxysmal or permanent AF, or atrial flutter, fast-rate or slow-rate AF. No additional referable information was provided for subsequent interventions for atrial fibrillation. Third, the mechanism underlying the increased risk of atrial fibrillation associated with the use of β-blockers is unclear and requires additional clinical or animal studies for confirmation. Fourth, whether β-blockers benefit certain populations, such as those with heart failure with a preserved or reduced ejection fraction, coronary heart disease, myocardial infarction, chronic obstructive pulmonary disease, or chronic kidney disease, is controversial. Further verification of whether beta-blockers increase the risk of atrial fibrillation in these populations is needed. Fifth, although our findings are based on analyses of almost the entire US population, whether our findings can be generalized to other races, ethnicities or countries remains uncertain and needs to be further explored.

## Conclusion

The results of this cohort study showed that β-blockers increase the risk of atrial fibrillation in cardiovascular-diseased patients. Similar results were observed in patients that were aged 65 years or older, male and female, non-Hispanic white people, hypertensive, diabetic, or hyperlipidemic. The findings may help physicians select a modest medication for patients while also assessing the risk of AF.

## Perspectives

### Competency in medical knowledge

β-blockers increase the risk of atrial fibrillation in cardiovascular-diseased patients; similar results were observed in patients that were aged 65 years or older, male and female, non-Hispanic white people, hypertensive, diabetic, or hyperlipidemic.

### Translational outlook

Although β-blockers may increase the risk of atrial fibrillation in patients with cardiovascular disease, further research is needed to determine whether they can affect the risk of atrial fibrillation in people with myocardial infarction, coronary heart disease, heart failure with ejection fraction preservation, or reduction.

## Data Availability

The original contributions presented in the study are included in the article/Supplementary Material, further inquiries can be directed to the corresponding author.
