# Supplementary material for: β-blockades and the risk of atrial fibrillation in patients with cardiovascular diseases
Source: Front Pharmacol. 2024 Jun 25;15:1418465. doi: 10.3389/fphar.2024.1418465 (PMC11232185; doi:10.3389/fphar.2024.1418465)
Supplement: Supplementary file 1 [file Table1.DOCX]

|  | Overall | AF | Non AF | P value |
| --- | --- | --- | --- | --- |
| Covariates | n=11110 | n=186 | n=10924 |  |
| Age (mean (SD)) | 62.00 [50.00, 72.00] | 78.50 [70.00, 80.00] | 62.00 [50.00, 71.00] | <0.001 |
| Male (%) | 5503 (49.5) | 100 (53.8) | 5403 (49.5) | 0.244 |
| Race (%) |  |  |  |  |
| Mexican American | 1224 (11.0) | 2 ( 1.1) | 1222 (11.2) | <0.001 |
| Other Hispanic | 1031 ( 9.3) | 8 ( 4.3) | 1023 ( 9.4) |  |
| Non-Hispanic White people | 4270 (38.4) | 154 (82.8) | 4116 (37.7) |  |
| Non-Hispanic Black people | 3078 (27.7) | 16 ( 8.6) | 3062 (28.0) |  |
| Other Race | 1507 (13.6) | 6 ( 3.2) | 1501 (13.7) |  |
| BMI (mean (SD)) | 20.89 [18.30, 24.20] | 21.56 [18.56, 24.53] | 20.84 [18.30, 24.20] | 0.38 |
| Obesity (%) | 756 ( 6.8) | 10 ( 5.4) | 746 ( 6.8) | 0.435 |
| Smoking (%) |  |  |  |  |
| Never | 5610 (50.5) | 103 (55.4) | 5507 (50.4) | 0.021 |
| Former | 3221 (29.0) | 60 (32.3) | 3161 (28.9) |  |
| Now | 2279 (20.5) | 23 (12.4) | 2256 (20.7) |  |
| Drinking (%) | 7372 (66.4) | 120 (64.5) | 7252 (66.4) | 0.593 |
| Insurance (%) |  |  |  |  |
| No | 426 ( 3.8) | 1 ( 0.5) | 425 ( 3.9) | <0.001 |
| Yes | 9884 (89.0) | 182 (97.8) | 9702 (88.8) |  |
| Other | 800 ( 7.2) | 3 ( 1.6) | 797 ( 7.3) |  |
| Employment (%) |  |  |  |  |
| Working at a job | 4386 (39.5) | 21 (11.3) | 4365 (40.0) | <0.001 |
| With business but not at work | 193 ( 1.7) | 0 ( 0.0) | 193 ( 1.8) |  |
| Looking for work | 241 ( 2.2) | 0 ( 0.0) | 241 ( 2.2) |  |
| Not working | 6281 (56.5) | 165 (88.7) | 6116 (56.0) |  |
| Other | 4386 (39.5) | 21 (11.3) | 4365 (40.0) | <0.001 |
| Diabetes (%) | 3750 (33.8) | 63 (33.9) | 3687 (33.8) | 0.973 |
| Hypertension (%) | 9816 (88.4) | 164 (88.2) | 9652 (88.4) | 0.938 |
| Hypercholesteremia (%) | 6121 (55.1) | 114 (61.3) | 6007 (55.0) | 0.087 |
| Taken medicine in past month (%) |  |  |  |  |
| Yes | 9666 (87.0) | 186 (100.0) | 9480 (86.8) | <0.001 |
| No | 1437 (12.9) | 0 ( 0.0) | 1437 (13.2) |  |
| Refused | 5 ( 0.0) | 0 ( 0.0) | 5 ( 0.0) |  |
| Other | 2 ( 0.0) | 0 ( 0.0) | 2 ( 0.0) |  |
| Medicines taken, No.  (median [IQR]) | 4.00 [2.00, 6.00] | 3.00 [1.00, 6.00] | 7.00 [5.00, 10.00] | <0.001 |
| β-blockers (%) | 2585 (23.3) | 111 (59.7) | 2474 (22.6) | <0.001 |
| CCB (%) | 2271 (20.4) | 42 (22.6) | 2229 (20.4) | 0.466 |
| ARB/ACEI (%) | 5322 (47.9) | 101 (54.3) | 5221 (47.8) | 0.078 |
| Diuretic (%) | 2349 (21.1) | 60 (32.3) | 2289 (21.0) | <0.001 |
| Statins (%) | 3524 (31.7) | 96 (51.6) | 3428 (31.4) | <0.001 |
| Insulin (%) | 1432 (12.9) | 37 (19.9) | 1395 (12.8) | 0.004 |
| Metformin (%) | 2414 (21.7) | 39 (21.0) | 2375 (21.7) | 0.8 |

STable 1. Baseline Characteristics of the AF and Non AF groups

Taken medicine in past month was defined as there have used or taken medication for which a prescription in the past 30 days.

Medicines taken was defined as the number of prescription medicines reported.

Abreviations: AF, atrial fibrillation; CCB, Calcium channel blockers; ARB, Angiotensin Receptor Blockers; ACEI, Angiotensin converting enzyme inhibitors.

|  | β-blockers | Non β-blockers | P value |  |
| --- | --- | --- | --- | --- |
| Covariates | n=1537 | n=3074 |  | Standardized difference |
| Age (mean (SD)) | 63.02 (12.46) | 63.89 (12.18) | 0.022 | 0.071 |
| Male | 733 ( 47.7) | 1462 ( 47.6) | 0.958 | 0.003 |
| Race (%) |  |  |  |  |
| Mexican American | 139 ( 9.0) | 287 ( 9.3) | 0.713 | 0.046 |
| Other Hispanic | 153 ( 10.0) | 283 ( 9.2) |  |  |
| Non-Hispanic White people | 624 ( 40.6) | 1234 ( 40.1) |  |  |
| Non-Hispanic Black people | 419 ( 27.3) | 888 ( 28.9) |  |  |
| Other Race | 202 ( 13.1) | 382 ( 12.4) |  |  |
| BMI (mean (SD)) | 21.60 (5.45) | 21.52 (5.22) | 0.647 | 0.014 |
| Obesity (%) | 109 ( 7.1) | 201 ( 6.5) | 0.519 | 0.022 |
| Smoking (%) |  |  |  |  |
| Never | 769 ( 50.0) | 1550 ( 50.4) | 0.181 | 0.058 |
| Former | 476 ( 31.0) | 884 ( 28.8) |  |  |
| Now | 292 ( 19.0) | 640 ( 20.8) |  |  |
| Drinking (%) | 1042 ( 67.8) | 2009 ( 65.4) | 0.106 | 0.052 |
| Insurance (%) |  |  |  |  |
| No | 40 ( 2.6) | 84 ( 2.7) | 0.897 | 0.015 |
| Yes | 1424 ( 92.6) | 2852 ( 92.8) |  |  |
| Other | 73 ( 4.7) | 138 ( 4.5) |  |  |
| Employment (%) |  |  |  |  |
| Working at a job | 525 ( 34.2) | 993 ( 32.3) | 0.476 | 0.058 |
| With business but not at work | 26 ( 1.7) | 41 ( 1.3) |  |  |
| Looking for work | 25 ( 1.6) | 46 ( 1.5) |  |  |
| Not working | 959 ( 62.4) | 1992 ( 64.8) |  |  |
| Diabetes (%) | 523 ( 34.0) | 996 ( 32.4) | 0.283 | 0.035 |
| Hypertension (%) | 1387 ( 90.2) | 2825 ( 91.9) | 0.067 | 0.058 |
| Hypercholesteremia (%) | 914 ( 59.5) | 1874 ( 61.0) | 0.343 | 0.031 |
| Taken medicine in past month (%) | 1537 (100.0) | 3074 (100.0) | NA | <0.001 |
| Medicines taken, No.  (median [IQR]) | 4.69 (2.20) | 4.28 (2.28) | <0.001 | 0.184 |
| CCB (%) | 385 ( 25.0) | 795 ( 25.9) | 0.575 | 0.019 |
| ARB/ACEI (%) | 883 ( 57.4) | 1774 ( 57.7) | 0.891 | 0.005 |
| Diuretic (%) | 323 ( 21.0) | 784 ( 25.5) | 0.001 | 0.106 |
| Statins (%) | 586 ( 38.1) | 1135 ( 36.9) | 0.445 | 0.025 |
| Insulin (%) | 195 ( 12.7) | 396 ( 12.9) | 0.889 | 0.006 |
| Metformin (%) | 360 ( 23.4) | 719 ( 23.4) | 1 | 0.001 |
| AF (%) | 36 ( 2.3) | 35 ( 1.1) | <0.001 |  |

STable 2. Baseline characteristics of β-blocker and non β-blocker groups after propensity score matching (1:2 ratio)

STabel 3. Diagnosis codes for identifying baseline comorbidities

| **Comorbidities** | ICD-10-CM codes |
| --- | --- |
| Hypertension | I10 |
| Diabetes | E11 |
| Hypercholesteremia | E78.0 |
| Atrial fibrillation | I48 |

| Population | Event no.(incidence rate) | Adjusted OR (95% CI) | P value |
| --- | --- | --- | --- |
| Age ≥65 |  |  |  |
| β-blockers group | 96/1561(6.1%) | 2.292 (1.547- 3.426) | <0.001 |
| No β-blockers group | 67/3159(2.1%) | 1 (ref.) |  |
| Age <65 |  |  |  |
| β-blockers group | 15/1024(1.4%) | 5.782 (2.167- 16.540) | 0.001 |
| No β-blockers group | 8/5366(0.1%) | 1 (ref.) |  |
| Non-hispanic white people |  |  |  |
| β-blockers group | 90/1198(7.5%) | 2.268 (1.520- 3.408) | <0.001 |
| No β-blockers group | 64/3.72(2.0%) | 1 (ref.) |  |
| Other races |  |  |  |
| β-blockers group | 21/1387(1.5%) | 4.171 (1.684- 11.326) | 0.003 |
| No β-blockers group | 11/5453(0.2%) | 1 (ref.) |  |
| Male |  |  |  |
| β-blockers group | 58/1266(4.5%) | 2.381 (1.449- 3.969) | 0.001 |
| No β-blockers group | 42/4237(0.9%) | 1 (ref.) |  |
| Female |  |  |  |
| β-blockers group | 53/1319(4.0%) | 2.203 (1.295- 3.785) | 0.004 |
| No β-blockers group | 33/4288(0.4%) | 1 (ref.) |  |
| Hypertension |  |  |  |
| β-blockers group | 98/2412(4.0%) | 2.271 (1.526- 3.407) | <0.001 |
| No β-blockers group | 66/7404(0.8%) | 1 (ref.) |  |
| No hypertension |  |  |  |
| β-blockers group | 13/171(7.5%) | 3.750 (2.039- 7.123) | <0.001 |
| No β-blockers group | 9/1121(0.8%) | 1 (ref.) |  |
| Diabetes |  |  |  |
| β-blockers group | 43/969(4.4%) | 2.555 (1.268- 5.365) | 0.001 |
| No β-blockers group | 20/2781(0.7%) | 1 (ref.) |  |
| No diabetes |  |  |  |
| β-blockers group | 68/1616(4.2%) | 2.231 (1.435- 3.485) | <0.001 |
| No β-blockers group | 55/5744(0.9%) | 1 (ref.) |  |
| Hypercholesteremia |  |  |  |
| β-blockers group | 67/1670(4.0%) | 2.221 (1.365- 3.651) | 0.001 |
| No β-blockers group | 47/4451(1.0%) | 1 (ref.) |  |
| No hypercholesteremia |  |  |  |
| β-blockers group | 44/915(4.8%) | 3.750 (2.039- 7.123) | <0.001 |
| No β-blockers group | 28/4074(0.6%) | 1 (ref.) |  |

STable 4. Detailed results of the analyses stratified by age, sex, race, and comorbidities

**SFig 1. Abstract graph**

**
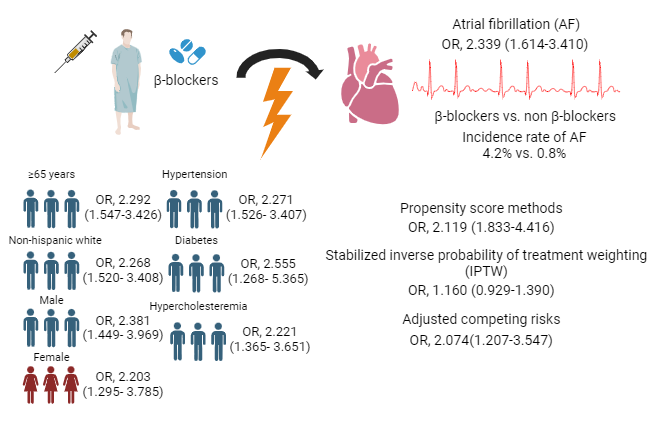
**

STable 5. Analysis of the relationship between type of β-blocker and AF

| β-blocker types | Adjusted OR | P value |
| --- | --- | --- |
| Metoprolol | 5.86(4.01-8.56) | <0.001 |
| Carvedilol | 4.99(3.03-8.22) | <0.001 |
| Atenolol | 2.27(1.15-4.47) | 0.018 |
| other | 3.89(2.15-7.04) | <0.001 |
| Non β-blocker | 1 [Reference] |  |
